# Inhibition of Melanoma Growth by Subcutaneous Administration of hTERTC27 Viral Cocktail in C57BL/6 Mice

**DOI:** 10.1371/journal.pone.0012705

**Published:** 2010-09-13

**Authors:** Longfei Huo, Hong Yao, Xicai Wang, Gee Wan Wong, Hsiang-fu Kung, Marie C. Lin

**Affiliations:** 1 Open Laboratory of Chemical Biology of the Institute of Molecular Technology, Department of Chemistry, The University of Hong Kong, Pokfulam, Hong Kong, China; 2 Institute of Pathology and Southwest Cancer Center, Southwest Hospital, Third Military Medical University, Chongqing, China; 3 Stanley Ho Center for Emerging Infectious Diseases, The Chinese University of Hong Kong, Shatin, Hong Kong, China; 4 Brain Tumor Center, Neurosurgery, Faculty of Medicine, The Chinese University of Hong Kong, Prince of Wales Hospital, Shatin, Hong Kong, China; 5 Tumor Hospital of Yunnan Province, Tumor Institute of Yunnan Province, Cancer Research Department of Biomedical Engineering Research Centre, Kunming Medical College, Kunming, China; Centre de Recherche Public de la Santé, Luxembourg

## Abstract

**Background:**

hTERTC27 is a 27 kDa C-terminal polypeptide of human telomerase reverse transcriptase that has previously been shown to reduce tumorigenicity of HeLa cells and suppress growth of xenografted glioblastoma in nude mice. Although ectopic expression of hTERTC27 upregulated genes that are involved in apoptosis, cell cycle, and immune response, the mechanism for hTERTC27-induced tumor suppression has not been completely elucidated. Since hTERT was identified as a universal tumor-associated antigen, we hypothesize that hTERTC27 inhibits tumor growth *in vivo* through activation of anti-tumor immune response.

**Methodology/Principal Finding:**

Immunocopetent C57BL/6 mice were used for mouse B16 melanoma model. Mice bearing B16 melanoma were administered rAAV-/rAdv viral cocktail expressing hTERTC27, and tumor growth was monitored after viral cocktail treatment. Blood and splenocytes were used to determine the level of cytokines and the activity of immune cells, respectively. B16 tumor growth was significantly inhibited by subcutaneous administration of a single dose of 1.5×10^11^ vg rAAV-hTERTC27 and 2.5×10^9^ pfu rAdv-hTERTC27 viral cocktail (rAAV-/rAdv-hTERTC27). The population and cytotoxicity of NK cells in the mice were significantly augmented by rAAV-/rAdv-hTERTC27 treatment, and selective depletion of the NK cell population in mice by intraperitoneal injection of anti-GM1 antibody abrogated the growth suppression of melanoma induced by rAAV-/rAdv-hTERTC27 administration.

**Conclusion:**

Activation of NK cells by administration of rAAV-/rAdv-hTERTC27 is critical for growth suppression of melanoma in mouse model.

## Introduction

The frequency of melanoma cases in Western countries has risen rapidly over the last years, and melanoma has become one of the most fatal cancers [1]. Local melanoma can be cured by wide surgical excision at its early stage [2], but metastatic melanomas are usually incurable [3]. Chemotherapy (e.g. dacarbazine) and cytokine adjuvant therapy (e.g. high-dose IFN-α2b and IL-2) are commonly used as a palliative systemic therapy in patients with advanced melanoma [4], [5], [6]. However, the non-specificity and significant side effects of these therapies greatly limit their effective use in patients [2], [7], [8], [9]. Biochemotherapy, a combination therapy of cytokine adjuvant with chemotherapeutic agents, has been shown to improve response rates but not overall survival. Moreover, biochemotherapy has been found to be associated with increased toxicity [10], [11]. Recently, several Phase II/III clinical trials are ongoing for the use of CTLA4 monoclonal antibodies as a new promising strategy for the treatment of metastatic melanoma [12]; however, significant autoimmune-related side effects have been increasingly observed [13], [14], [15]. Cancer vaccines, on the other hand, exhibit higher specificity and less toxicity, and their development has made rapid progress in recent decades [10], [16], [17], but the therapeutic efficacy has been very low with the reported overall objective response rate of only 3.3% [18].

Telomeres are specialized structures at the end of eukaryotic chromosomes that function to prevent chromosome end-joining and maintained by telomerase, a ribonucleoprotein complex that functions in elongating telomeres using reverse transcriptase and a specific RNA molecule in the complex. Telomere length loss occurs with cell division in somatic cells in which telomerase activity is absent and induces replicative senescence and cell proliferation inhibition when the length decreases to below a certain threshold [19]. In contrast, immortal cells like stem cells and cancer cells express high telomerase activity and show little loss of telomere length with cell division, and thus escape replicative senescence and proliferate indefinitely [20]. Telomerase reverse transcriptase (TERT), the catalytic peptide subunit of telomerase, is expressed in more than 85% of human tumor cells but rarely in normal cells, making it an ideal target for antigen-specific cancer immunotherapy [21], [22]. Indeed, studies have shown that TERT able to trigger antitumor cytotoxic T lymphocyte (CTL) responses, and immunization of mice with TERT stimulated TERT-specific CTL that can kill cancers of various origins [23], [24], [25].

hTERTC27 is an artificially derived 27 kD C-terminal polypeptides of human TERT [26]. Overexpression of hTERTC27 in HeLa cells caused excessive chromosome end joining events without affecting telomerase activity in TRAP assay [26]. Previously, we developed a novel cancer gene therapy using recombinant adeno-associated virus (rAAV) as delivery vector for hTERTC27 (i.e. rAAV-hTERTC27). In a glioblastoma xenograft mouse model, we observed that ectopic expression of hTERTC27 in tumor cells induced tumor regression and significantly prolonged survival of tumor-bearing mice. The action of hTERTC27 on tumors is mechanistically complex. For instance, transduction of tumor cells with rAAV-hTERTC27 induced cell apoptosis and inhibited tumor angiogenesis. Moreover, we observed an influx of ploymorphonuclear neutrophils into hTERTC27-treated tumor xenograft, and upregulation of gene expression involved in immune response [27], suggesting that the immune response might play a role in tumor regression.

Previously, it has been reported that administration of adeno-associated virus (rAAV) with adenovirus (rAdv) together can increase AAV transduction efficiency by >100 fold [28]. For example, the rAAV-BMP2-induced osteogenic activity was successfully enhanced by combination of rAAV-BMP2 with a low level of rAdv-BMP2 [29]. Similarly, a low level of rAdv-hTERTC27 greatly enhanced the expression of hTERTC27 transgene carried by rAAV, and rAAV-/rAdv-hTERTC27 viral cocktail potently inhibited xenografted glioblastoma growth [30].

In this study we tested the efficacy of the rAAV-/rAdv-hTERTC27 viral cocktail in treating melanoma and explored the possible involvement of immune response in cancer regressions mediated by rAAV-/rAdv-hTERTC27 treatment using an immunocompetent mouse model of melanoma. We found that a single dose administration of viral cocktail of hTERTC27 was sufficient to significantly inhibit the tumor growth of melanoma in C57BL/6 mice. The innate arm of immunity mediated by NK cells and adaptive immunity mediated by Th1 cytokines seemed to play an important role in this anti-tumor effect. The potential beneficial effects of supplementing rAAV-/rAdv-hTERTC27 gene therapy with other therapies for prevention and treatment of melanoma metastasis warrant further investigations.

## Materials and Methods

### Animals, cells, antibodies and reagents

Female C57BL/6N mice (6–8 weeks old) were purchased from the Charles River Laboratories (Wilmington, MA) and housed under aseptic conditions and cared for according to the guidelines issued by the University of Hong Kong's Laboratory Animal Unit. Mice were fed for three days before the experiments. All experimental protocols were approved by the Department of Health of the Government of HKSAR [permit number: (411) in DH/ORHI/8/2/3 Pt. 5] and the University of Hong Kong's Committee on the Use of Live Animals in Teaching and Research (approval ID: CULATR 1334–06).

Mouse melanoma B16-F1 (ATCC, CRL-6323) cells were cultured in DMEM supplemented with 10% heat inactivated FBS, 100 U/ml penicillin and 100 µg/ml streptomycin (Invitrogen, Carlsbad, CA) at 37°C with 5% CO_2_. YAC-1 (ATCC, TIB-160) cells were maintained in RPMI-1640 medium supplemented with 10% heat inactivated FBS and antibiotics (100 U/ml penicillin & 100 µg/ml streptomycin).

CytoTox 96 Non-Radioactive Cytotoxicity Assay and CellTiter 96 Non-Radioactive Cell Proliferation Assay Kit were purchased from Promega (Promega, WI, USA). Mitomycin C, Lipopolysaccharides (LPS) from *Escherichia coli* O111:B4, Concanavalin A (ConA), and Histopaque®-1083 were obtained from Sigma (Sigma, MO, USA). Red blood cell lysis buffer, recombinant mouse IL-2, R-Phycoerythrin (R-PE)-conjugated rat anti-mouse CD4 monoclonal antibody (L3T4), Fluorescein Isothiocyanate (FITC)-conjugated rat anti-mouse CD8a monoclonal antibody (Ly-2), R-PE-conjugated rat anti-mouse CD49b/Pan NK monoclonal antibody (DX5), FITC-conjugated rat anti-mouse CD3 monoclonal antibody (17A2), FITC-conjugated rat anti-mouse CD19 monoclonal antibody (1D3) were all obtained from BD Biosciences (BD Bioscience Pharmingen).

### Production of recombinant adenovirus (rAdv) and adeno-associated virus (rAAV)

Recombinant adenovirus-hTERTC27 (rAdv-hTERTC27) and recombinant adeno-assoicated virus-hTERTC27 (rAAV-hTERTC27) were prepared as previously described [30], and the recombinant viruses were kept at −80°C prior to use.

### Subcutaneous tumor inoculation

Melanoma B16 cells were harvested in exponential growth phase by trypsinization and washed twice with ice-cold PBS, and then resuspended into ice-cold PBS at a concentration of 2×10^6^ cells/ml. C57BL/6 mice were subcutaneously injected with B16 cells (2×10^5^ cells/mouse) on the right back flank. 7 days later, each mouse with tumor about 0.2∼0.4 cm^3^ received a viral cocktail injection (1.5×10^11^ vg of rAAV-hTERTC27 plus 2.5×10^9^ pfu of rAdv-hTERTC27 for treatment groups; or 1.5×10^11^ vg of rAAV-EGFP plus 2.5×10^9^ pfu of rAdv-EGFP for viral cocktail control group) or equal volume of PBS injection (for control group) by routes as indicated. Tumor size was measured with calipers every other day. Tumor volume (*V*) was calculated by the formula, *V* = 1/2×*S*×S×*L*, where *S* and *L* are the shortest and longest diameter of the tumor, respectively. Mice were sacrificed on day 21 post-tumor cell injection.

### Profile study of lymphocytes in blood by flow cytometry

Peripheral blood was collected from each mouse at sacrifice and mixed with anticoagulant immediately. Plasma was collected by centrifugation at 300 ×g for 5 min, and the cell pellet was washed twice with PBS and resuspended in 0.4 ml of PBS-FBS (PBS, pH 7.4, containing 1% FBS and 0.1% sodium azide). The cell suspension was divided into two parts with equal volume. One part was labeled with PE-conjugated rat anti-mouse CD4 monoclonal antibody and FITC-conjugated rat anti-mouse CD8 monoclonal antibody, while the other part was labeled with PE-conjugated rat anti-mouse CD49b/pan-NK monoclonal antibody and FITC-conjugated rat anti-mouse CD19 monoclonal antibody, for 40 min on ice. For detection of NKT cells, plasma was labeled with PE-conjugated rat anti-mouse CD49b monoclonal antibody and FITC-conjugated rat anti-mouse CD3 monoclonal antibody. After red blood cell lyses and washing, the cells were analyzed by Epics Altra (Bechman Coulter, Miami, FL) and free software WinMDI version 2.8 (http://facs.scripps.edu/software.html).

### Depletion of NK cell population in mice

To deplete NK cell population, mice were administered 20 µl of anti-asialo GM1 (Wako Pure Chemical Industries) by intraperitoneal injection 3 days before viral cocktail treatment, followed by repeated injection every five days for duration of two weeks. NK cell population in blood was then analyzed by flow cytometry.

### Measurement of cytokine levels

Cytokine levels in plasma collected from mouse peripheral blood were analyzed using T_H_1/T_H_2 10plex kit and FlowCytomix Pro 1.0 Software (Bender MedSystems, USA) according to the manufacturer's instructions.

### Lymphocyte proliferation activity

Lymphocytes were isolated from splenocytes by HistoPaque-1083 centrifugation and then seeded into 96-well plate with 100 µl per well at the concentration of 5×10^6^ cells/ml. After 72 hrs stimulation with 10 µg/ml ConA or 10 µg/ml LPS, the lymphocyte proliferation activity was analyzed using CellTiter 96 Non-Radioactive Cell Proliferation Assay Kit and the activity of LDH was measured by a spectrophotometer at 490 nm. The stimulation index (SI) was calculated as the formula SI  =  (Mitogen treatment-Background)/(Control-Background).

### Cytotoxic activities of NK and CTL

The cytolytic activities of NK and CTL were determined by CytoTox 96 Non-Radioactive Cytotoxicity Assay Kit. Briefly, for NK cytotoxic activity, lymphocytes isolated from spleen of each mouse were incubated with YAC-1 or B16 cells at different effector/target (E:T) ratios at 37°C for 4 hrs, and targeted cell lysis was calculated according to the manufacturer's instructions. For CTL activity, lymphocytes isolated from spleen of each mouse were stimulated with mitomycin C-treated B16 cells (a 2∶1 ratio of lymphocytes and B16) and recombinant mouse IL-2 (mIL-2, 50 ng/ml) in complete culture medium for six days. Culture medium (half of volume) was replenished with fresh medium containing mIL-2 every 3 days. Viable lymphocytes were separated by Histopaque-1083 density gradient centrifugation and then incubated with B16 cells at the indicated E:T ratio at 37°C for 4 hrs. Cytotoxicity was measured using CytoTox 96 Non-Radioactive Cytotoxicity Assay Kit.

### Statistical analysis

Student's *t* test was used to compare tumor volumes and cytotoxicity between experimental groups. Prism 3.0 (GraphPad Software, San Diego California, USA) was used for all calculations, with a *P*<0.05 deemed statistically significant.

## Results

### rAAV-/rAdv-hTERTC27 viral cocktail is more effective than either virus alone in suppressing the growth of B16 melanoma in C57BL/6 mice

**Figure 1:**
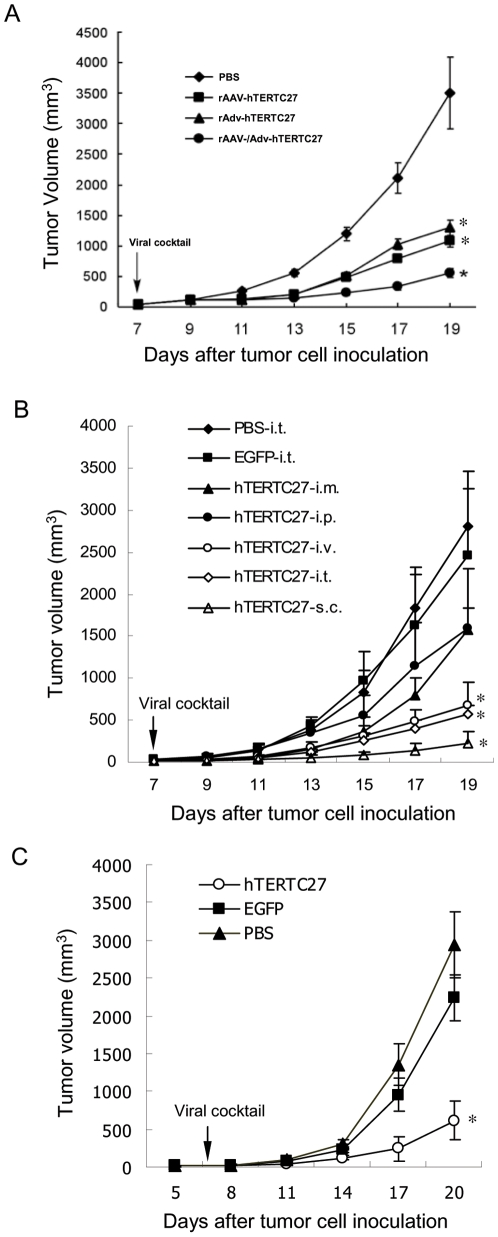
rAAV-/rAdv-hTERTC27 treatment suppresses the growth of B16 melanoma in C57BL/6 mice. (A) Anti-tumor efficacy of rAAV-hTERTC27, rAdv-hTERTC27 and rAAV-/rAdv-hTERTC27. Tumor-bearing mice (5mice/group) were subcutaneously injected with viruses as indicated. Tumor growth was monitored by measuring tumor size with calipers every other day till the day when mice sacrificed. *: *P<0.05*, compared with PBS treated group. (B) Antitumor efficacies of different administration routes of rAAV-/rAdv-hTERTC27 viral cocktail. Tumor-bearing mice were treated by injection of rAAV-/rAdv-hTERTC27, rAAV-/rAdv-EGFP, or PBS as indicated routes, i.e. intratumor (i.t.), intra-muscular (i.m.), intraperitoneal (i.p.), intravenous (i.v.) and subcutaneous (s.c.) injection (3mice/group). *: *P<0.05*, compared with PBS treated group. (C) Antitumor efficacy of subcutaneous administration of hTERTC27. Melanoma-bearing mice were subcutaneously received rAAV-/rAdv-hTERTC27 viral cocktail (5 mice), rAAV-/rAdv-EGFP viral cocktail (4 mice) or PBS (3 mice) around tumor on 7^th^ day after tumor cell injection, and were sacrificed on day 20 post-tumor cell injection. *: *P<0.05*, compared with PBS group.

We first tested the efficacy of the rAAV-/rAdv-hTERTC27 viral cocktail in a melanoma cancer model. C57BL/6 mice received subcutaneous injection of PBS, rAAV-/rAdv-hTERTC27 viral cocktail or either virus alone near the tumors on the 7th day after tumor cell inoculation. As expected, tumors in control mice treated with PBS grew much more rapidly than those in mice treated with either rAAV-hTERTC27 or rAdv-hTERTC27 or both over the observation period (Fig. 1A). Moreover, administration of rAAV-/rAdv-hTERTC27 viral cocktail significantly suppressed tumor growth compared with either virus alone (Fig. 1A).

We then investigated the efficacy of administration routes of rAAV-/rAdv-hTERTC27 in melanoma growth. Mice bearing B16 melanoma were given a single dose of rAAV-/rAdv-hTERTC27 by different routes (intratumoral, intramuscular, intravenous, intraperitoneal, and subcutaneous) or rAAV-/rAdv-EGFP (intratumoral injection), or PBS (intratumoral injection). Tumor growth in mice received intraperitoneal or intramuscular injection of rAAV-/rAdv-hTERTC27 viral cocktail was not significantly different from the control group mice, which received an intratumoral injection with either PBS or rAAV-/rAdv-EGFP viral cocktail (Fig. 1B). However, we observed much slower tumor growth in mice that received intratumoral, intravenous or subcutaneous injection of rAAV-/rAdv-hTERTC27 viral cocktail compared with mice from control groups (Fig. 1B). Unexpectedly, subcutaneous injection of rAAV-/rAdv-hTERTC27 viral cocktail around tumor resulted in the most significant tumor-growth inhibition (Fig. 1B). Consistent results were obtained from another experiment in which mice were injected subcutaneously with rAAV-/rAdv-hTERTC27, rAAV-/rAdv-EGFP or PBS. Again, mice that received rAAV-/rAdv-hTERTC27 exhibited greater tumor growth inhibition than those given rAAV-/rAdv-EGFP or PBS (Fig. 1C).

### NK cells population and cytotoxicity are enhanced by administration of rAAV-/rAdv-hTERTC27 viral cocktail

**Figure 2:**
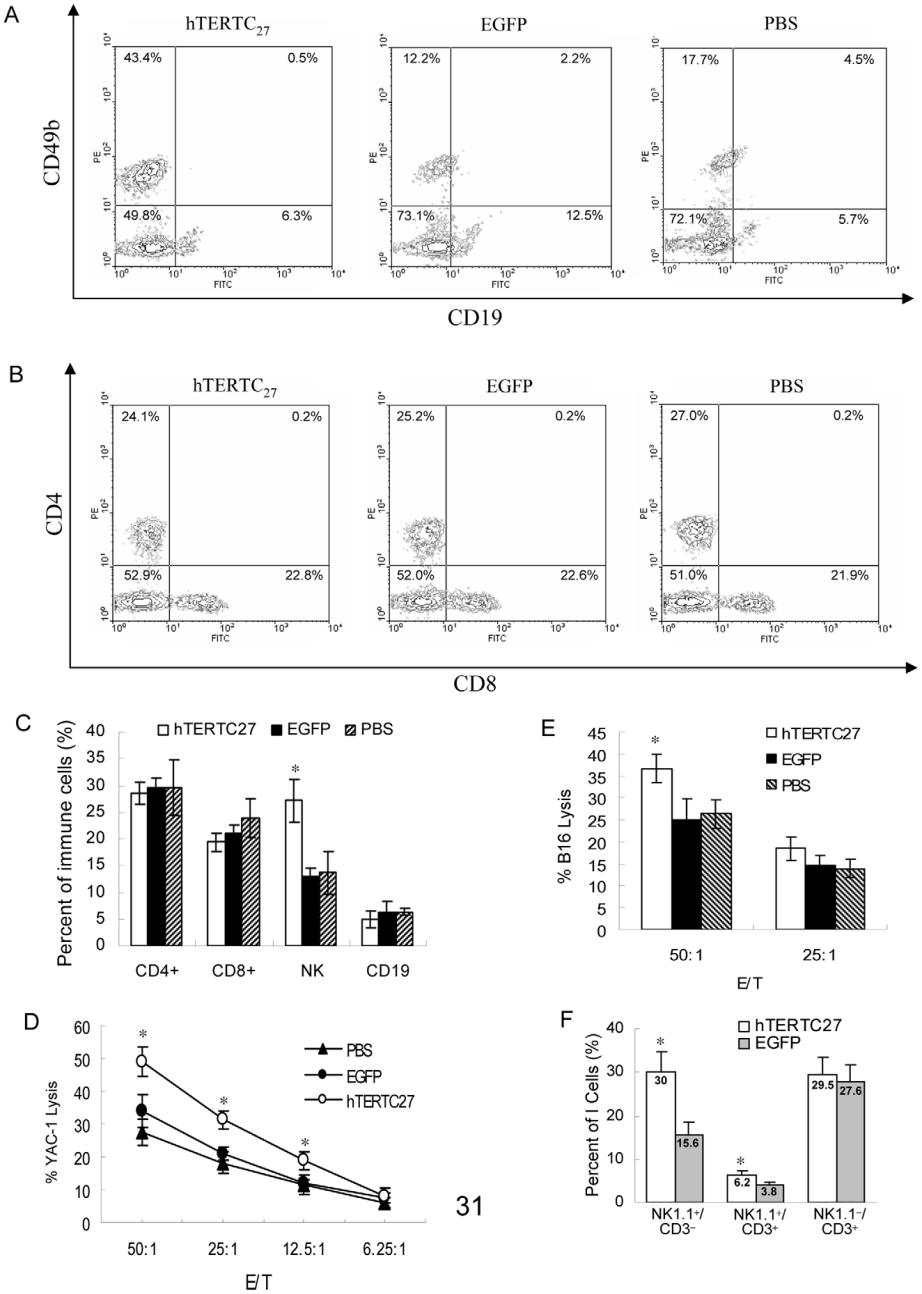
rAAV-/rAdv-hTERTC27 treatment increases the population and cytotoxicity of NK cells. (A) & (B) Flow cytometric analysis of PMBC from tumor-bearing mice treated with subcutaneous administration of rAAV-/rAdv-hTERTC27, rAAV-/rAdv-EGFP, or PBS. Data shown here represent one of several mice analyzed with similar results. (C) Lymphocyte profiles from A & B (data pooled from 3 mice/group treated with indicated viruses or PBS). *P<0.05, compared with EGFP group. (D) and (E): Cytotoxic activity of spleen NK cells isolated from mice treated with rAAV-/rAdv-hTERTC27, rAAV-/rAdv-EGFP or PBS was analyzed against YAC-1 (D) or B16 (E) cells. Lymphocytes isolated from each mouse were cultured with YAC-1 or B16 cells at various effector to target ratios (E/T). *: *P<0.05*, compared with EGFP group. (F): Profiles of NKT (NK1.1^+^/CD3^+^), NK (NK1.1^+^/CD3^−^) and T (NK1.1^−^/CD3^+^) cells in PMBC from tumor-bearing mice treated with rAAV-/rAdv-hTERTC27 or rAAV-/rAdv-EGFP. The numerical value presented with the bar graph indicates the percentage of the cell population in PMBC. The cell population without any marker (i.e. NK1.1^−^/CD3^−^) is not shown. Data pooled from 3 mice/group treated with indicated virus through flow cytometry analysis. *: *P<0.05*, compared with EGFP group.

Our previous study indicated that in addition to apoptosis genes, rAAV-hTERTC27 also regulates the expression of genes that are important to immune functions [27]. To test the hypothesis that an immune response is one of the major mechanisms responsible for tumor suppression executed by rAAV-/rAdv-hTERTC27, we analyzed the population profiles of peripheral blood leukocytes by flow cytometry. The population of NK cells was significantly increased in mice treated by rAAV-/rAdv-hTERTC27 viral cocktail (43.4%) compared with mice treated by rAAV-/rAdv-EGFP (12.2%) or PBS (17.7%) (Fig. 2A and 2C). However, there was no significant difference in the populations of B cells, CD4^+^ T cells and CD8^+^ T cells between the different treatment groups (Fig. 2B and 2C). The antibody used for pan NK cell (NK1.1^+^) profile study is an anti-CD49b antibody (DX5) which also recognizes NKT (NK1.1^+^/CD3^+^) cells. To clarify whether the population of NKT cells is increased by the rAAV-/rAdv-hTERTC27 treatment, another flow cytometry analysis was performed using PE-conjugated rat anti-mouse CD49b and FITC-conjugated rat anti-mouse CD3 antibodies. As shown in Figure 2F, the population of NK (NK1.1^+^/CD3^−^) cells was consistently increased by hTERTC27 treatment. In addition, hTERTC27 increased the population of NKT (NK1.1^+^/CD3^+^) cells but did not significantly change the population of NK1.1^−^/CD3^+^ T cells (Fig 2F). Although the populations of both NK (NK1.1^+^/CD3^−^) cells and NKT (NK1.1^+^/CD3^+^) cells were significantly increased by hTERTC27, the increase in NK cell population (192%) is higher than NKT cell population (159%). Moreover, the percentage of NK cells (30%) in PMBC is much higher than that of NKT cells (6%) (Fig 2F). These results suggest that NK (NK1.1^+^/CD3^−^) cells may play much more important roles than NKT cells (NK1.1^+^/CD3^+^) in suppressing xenografted tumor growth.

To further investigate whether administration of rAAV-/rAdv-hTERTC27 viral cocktail can increase the cytolytic activity of NK cells, NK cell cytotoxicity assay was performed. Lymphocytes from mice that received rAAV-/rAdv-hTERTC27 injection exhibited a significant increase in YAC-1 (a target cell of NK) killing activity as compared to those from mice treated with rAAV-/rAdv-EGFP or PBS at the E/T ratios of 50∶1 and 12.5∶1, respectively (Fig. 2D). In addition, lymphocytes from mice treated with rAAV-/rAdv-hTERTC27 also exhibited higher cytotoxicity to B16 cells than those from mice treated with rAAV-/rAdv-EGFP or PBS at the E/T ratio of 50∶1 (Fig. 2E).

### Suppression of NK cell impairs the antitumor effects of rAAV-/rAdv-hTERTC27 administration

**Figure 3:**
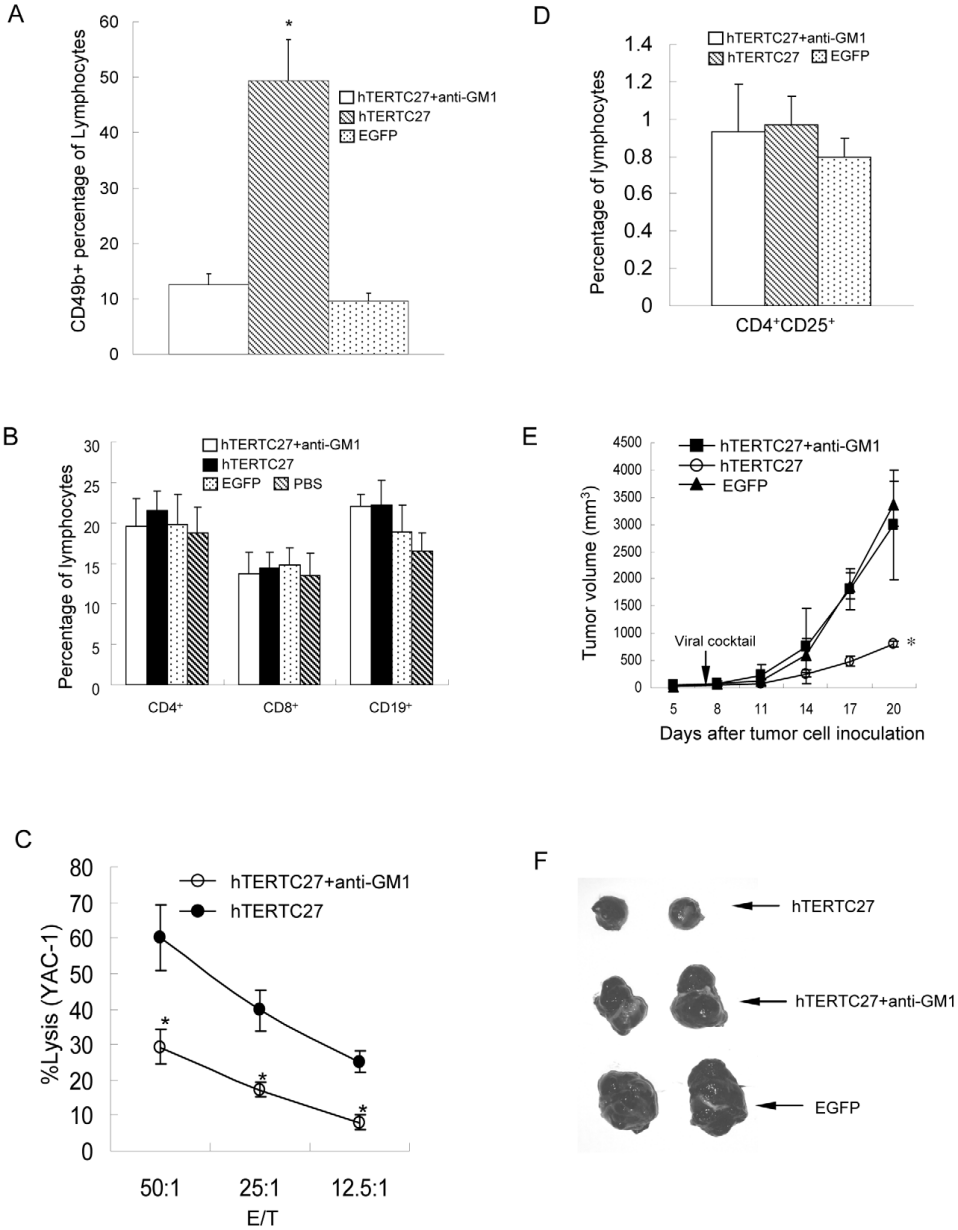
NK cell activity is crucial for the anti-tumor effect of hTERTC27. PMBC from mice intraperitoneally injected with anti-GM1 antibody were analyzed for populations of NK cells (A), helper T cell/cytotoxic T cell/B cell (B) and regulatory T cell (D) by flow cytometry. (C) Cytotoxicity of NK cells against YAC-1. Lymphocytes isolated from spleen of each mouse were cultured with YAC-1 cells at various E/T ratios, and the specific lysis was determined using CytoTox 96 Non-Radioactive Cytotoxicity Assay Kit. (E) NK cell depletion abrogated the anti-tumor effects of rAAV-/rAdv-hTERTC27 in C57BL/6 mice. *: *P<0.05*, compared with EGFP group. (F) A representative photo of tumor morphology from different treatment groups. hTERTC27: hTERTC27 viral cocktail; EGFP: EGFP viral cocktail. *: *P<0.05*, compared with hTERTC27 group.

The remarkable increase in the population and cytotoxicity of NK cells in hTERTC27 treated mice strongly hinted that NK cell is crucial for hTERTC27-induced tumor suppression. To test this hypothesis, NK cell population was depleted in mice by intraperitoneal injection of anti-GM1 antibody, and its effect on tumor growth during the rAAV-/rAdv-hTERTC27 treatment was investigated. As shown in Figure 3A, injection of anti-GM1 antibody specifically reduced NK cell population in PBMC, whereas helper T cells (CD4^+^), cytotoxic T cells (CD8^+^), B cells (CD19^+^) (Fig. 3B), and regulatory T cells were unaffected (Fig. 3D). Consistently, the cytotoxicity of splenocytes to YAC-1 was decreased in anti-GM1 antibody-treated mice (Fig. 3C). Strikingly, anti-GM1 antibody injection completely abolished the tumor growth suppression induced by hTERTC27 treatment (Fig. 3E, 3F). Taken together, the results suggest that NK cell activation is crucial for the anti-tumor effect of hTERTC27.

### T_H_1 cytokine secretion is increased after administration of rAAV-/rAdv-hTERTC27

**Figure 4:**
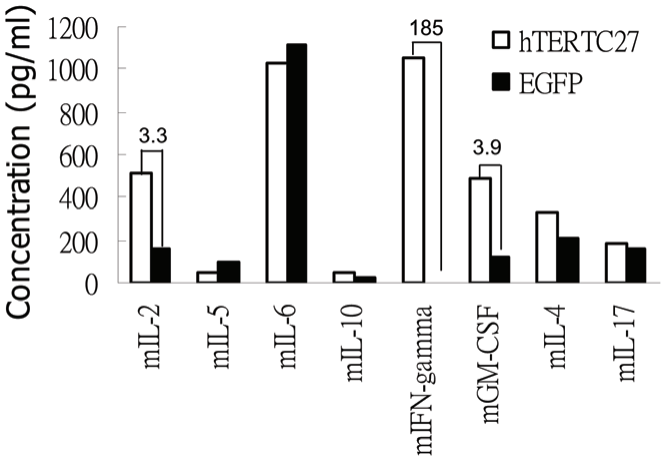
rAAV-/rAdv-hTERTC27 treatment increases plasma levels of Th1 cytokines. Plasma from sacrificed mice on day 20 post tumor cell inoculation were analyzed to determine cytokine levels using mouse Th1/Th2 FlowCytomix assay kit. The numerical values presented with the bar graph indicate the fold differences of cytokine levels between mice treated with hTERTC27 and EGFP.

To investigate blood cytokine levels, plasma from mice treated with rAAV-/rAdv-hTERTC27 and rAAV-/rAdv-EGFP viral cocktails was collected and analyzed using Mouse Th1/Th2 FlowCytomix kit. T_H_1 and T_H_2 cytokines refer to the patterns of cytokines secreted by two different subpopulations of CD4^+^ T cells that determine the outcome of an antigenic response toward humoral and cell-mediated immunity, respectively. Th1 cytokines include IL-2, IFN-γ, IL-12 and TNF-β, and Th2 cytokines include IL-4, IL-5, IL-6, IL-10 and IL-13. The levels of mIL-2, mIFN-γ, and mGM-CSF in blood of mice treated with hTERTC27 were 3.3-, 185- and 3.9-fold, respectively, higher than those in the blood of mice treated with EGFP. We did not observe a significant change in the level of other cytokines (e.g. IL-4, IL-5, IL-6, IL-10 and IL-17) between hTERTC27-treated mice and EGFP-treated mice (the level difference is <2-fold) (Fig.4).

### T cell activity is enhanced by administration of rAAV-/rAdv-hTERTC27

**Figure 5:**
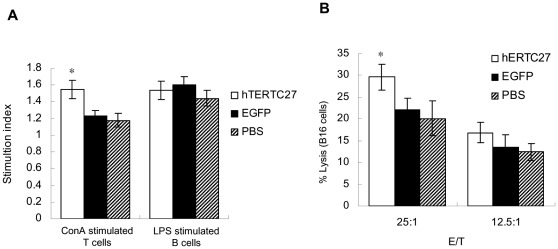
rAAV-/rAdv-hTERTC27 treatment has different effects on the activities of T cells and B cells. (A) Proliferation activity of T cells and B cells isolated from mice treated with hTERTC27, EGFP or PBS. Data are expressed as means ± SD (n = 3). *: *P*<0.05, compared with EGFP group. (B) Cytotoxicity of T cells isolated from mice treated with hTERTC27, EGFP, or PBS. *: *P*<0.05, compared with EGFP group.

Lymphocytes from spleen were treated with ConA (10 µg/ml) or LPS (10 µg/ml) to investigate whether administration of hTERTC27 viral cocktail can increase the proliferation activity of T cells or B cells. As shown in Figure 5A, there is no significant difference in B cell proliferation activity among groups treated with rAAV-/rAdv-hTERTC27, rAAV-/rAdv-EGFP or PBS. However, a slight but significant increase in T cell proliferation was observed in mice treated with rAAV-/rAdv-hTERTC27 as opposed to those treated with rAAV-/rAdv-EGFP or PBS. Consistent with the T cell proliferation assay, the cytotoxicity of spleen T lymphocytes was a little higher in mice treated with rAAV-/rAdv-hTERTC27 than in those treated with rAAV-/rAdv-EGFP or PBS (Fig. 5B).

## Discussion

In this study, we demonstrated for the first time that rAAV-/rAdv-hTERTC27, a viral cancer therapy effective in treating glioblastoma [30], also provides therapeutic benefit against murine melanoma. Our data suggest that rAAV-/rAdv-hTERTC27 acts through enhancing anti-tumor innate immunity.

The rationale of combining rAAV with rAdv vectors is that a non-therapeutic dose of rAdv-null can greatly increase the transduction level of rAAV, and a combination of therapeutic dose of rAdv-Luc (2.5×10^9^ pfu) and rAAV-Luc (1.5×10^11^ v.g.) dramatically increased the luciferase expression level *in vivo* [29], [30]. Therefore, a better beneficial antitumor effect was achieved by combining rAAV-hTERTC27 with rAdv-hTERTC27 compared with either one alone, suggesting that the enhanced expression level of hTERTC27 likely played a role.

As a universal tumor-associated antigen, TERT is an ideal target for cancer therapy. Conceptually, three approaches have exploited TERT for cancer therapy: gene therapy (using viral gene transfer to interfere telomerase activity or express suicide gene under TERT promoter), immunotherapy (stimulating TERT-specific immune response to kill TERT-expressing cells), and small-molecule inhibitors (using peptide/chemical drugs to inhibit telomerase activity) [31]. Previously, rAAV-hTERTC27 was developed as a TERT-targeting gene therapy [27]. Here, investigation of the mechanism by which rAAV-/rAdv-hTERTC27 inhibits melanoma led us to believe that one of the effects of rAAV-/rAdv-hTERTC27 might be the enhanced cancer immunosurveillance mediated mainly by NK cells since 1) a single dose administration of rAAV-/rAdv-hTERTC27 not only increased the population of blood NK cells by about two fold (Fig. 2) but also enhanced the specific cytotoxicity of splenic NK cells to YAC-1 and B16 cells (Fig. 5); 2) selective depletion of NK cells which are known to play important roles in tumor immunosurveillance *in vivo* [32] but not NKT cells by the anti-GM1 antibody [33]–[36] completely abrogated the antitumor effects of hTERTC27 (Fig. 3E). Although the population of NKT cells in PMBC was also increased by rAAV-/rAdv-hTERTC27 (Fig. 2F), the percentage of the NKT cells (6%) was too low as compared to that of NK cells (30%) to be the major effector cells which can induce such significant tumor growth suppression.

Our study indicates that activation of NK cells by hTERTC27 is sufficient to inhibit melanoma growth in mouse model, pinpointing the importance of NK cell activation in cancer immunotherapy. These observations are consistent with our previous study that rAAV-hTERTC27 administration effectively inhibited glioblastoma growth in nude mice, where T cells are absent but NK cells are functional. Consistently, a previous study also indicated that activation of NK cells can provide effective innate immunotherapy of melanoma in mouse model [37].

The NK activity-stimulating effect of rAAV-/rAdv-hTERTC27 represents a novel way to exploit the functions of TERT in cancer treatment. hTERTC27 contains two peptides, p973 and p988, both of which have been shown to induce TERT specific CTLs *in vitro* or *in vivo* to lyse TERT^+^ tumor cells, including melanoma B16 cells [38], [39]. It has also been reported that dendritic cells (DCs) pulsed with peptide p540 can elicit CTLs *ex vivo* to lyse hTERT^+^ tumor cells, including human melanoma cells K029 [25]. DCs transfected with mTERT gene can also induce CTLs to lyse B16 cells and inhibit B16 melanoma metastasis [40]. Apparently, the mechanism of hTERTC27-induced tumor suppression in our study is different from that of peptides (p973, p988, p540) or TERT gene transfected DCs reported in other studies. hTERTC27 may contain other unknown epitopes that have a tendency to induce a strong innate immune response other than an adaptive immune response. Not surprisingly, a single-peptide epitope (e.g. p988 or p973) tends to induce adaptive immunity because such single peptide is screened by CTL assay [38], [39]. In fact, some other epitopes which induce activation of CD4^+^ T cells but not CD8^+^ T cells from hTERT have been identified [41], [42], suggesting that some epitopes that specifically induce other immune cells may also exist. Thus, further study to identify the epitopes of hTERTC27 will likely be crucial to dissect the mechanism of hTERTC27-induced NK cell activation *in vivo*.

rAAV-/rAdv-hTERTC27 significantly inhibited B16 tumor growth when administered by intravenous, intratumor and subcutaneous injection. However, subcutaneous injection of rAAV-/rAdv-hTERTC27 viral cocktail near tumor site produced the most significant antitumor effect. Although it is unclear what might have contributed to the differences of antitumor effect among these administration routes, it has been reported that the induction of immunity (humoral, cell-mediated, or both) against the transgene product carried by rAAV depends on the routes of administration [43]. Therefore, intratumoral, intravenous or subcutaneous administration of rAAV-/rAdv-hTERTC27 may preferentially enable the stimulation of innate anti-tumor immunity. It will be interesting to investigate the effect of different administration routes on NK cell population and activation. Our observation implies that careful selection of administration route may be important for cancer gene therapy.

The population of T cells (CD4^+^ and CD8^+^) and B cells in blood did not vary significantly between the mice treated with rAAV-/rAdv-hTERTC27 and those treated with rAAV-/rAdv-EGFP or PBS (Fig. 2). Although small differences in T cell proliferation activity and CTL cytotoxicity were observed between hTERTC27 treated mice and control group mice (Fig. 5), they were mild and unlikely to be the major determinants responsible for the significant tumor suppression induced by rAAV-/rAdv-hTERTC27 observed in our study. A minor increase of CTL cytotoxicity in spleen lymphocytes might be a result of increased levels of Th1 cytokine in blood. A considerable increase in the levels of IL-2, IFN-γ and GM-CSF was observed in the plasma of mice treated with rAAV-/rAdv-hTERTC27 compared with the control mice (Fig. 4). All these cytokines function as an immune adjuvant and are known to contribute to the development and activity of tumor specific CTL [40], [42], [44]. However, further investigation is required to determine whether cytokines such as IL-2 and IFN-γ could increase the population of cytokine-induced-killer cells (CIK) [45] *in vivo* and contribute to the antitumor effects of hTERTC27. Unlike our previous study in which ectopic expression of rAAV-hTERTC27 in nude mice significantly up-regulated the IL-17 mRNA level [27] in xenografted tumor tissue, administration of rAAV-/rAdv-hTERTC27 viral cocktail in C57BL/6 mice showed a little increase of IL-17 cytokine level in blood in this study. The discrepancy may come from the different mouse models and delivery systems used between these two studies. Nonetheless, the slight change in IL-17 level is consistent with the mild increase in activated T cells because it is known that IL-17 expression is restricted to activated T-cells [46].

It is worth noting that NK cells are known to play a major role in cytokine-mediated inhibition of B16 melanoma development [47]–[51] and that IL-2 can induce the proliferation and activity of NK cells [48], [52]–[54]. Moreover, activated NK cells can secrete several cytokines, including IFN-γ and GM-CSF [55] and increase IL-2 mRNA expression [56]. In addition, IFN-γ itself also activates NK cells [57]. Because of the complex relationship between NK cells and these cytokines, the initial effect following hTERTC27 administration remains elusive and requires further investigation.
